# Surgical Prevention of Breast Cancer-Related Lymphedema: A Scoping Review

**DOI:** 10.3390/lymphatics3030015

**Published:** 2025-06-20

**Authors:** Shahnur Ahmed, Angad Sidhu, Luci Hulsman, Chilando M. Mulenga, Aladdin H. Hassanein

**Affiliations:** Division of Plastic Surgery, Indiana University School of Medicine, 545 Barnhill Drive, Indianapolis, IN 46202, USA;

**Keywords:** lymphedema, immediate lymphatic reconstruction, lymphovenous anastomosis, LYMPHA, lymph node transfer

## Abstract

Breast cancer-related lymphedema (BCRL) is the most common cause of secondary lymphedema in the Western world and occurs in up to one-third of breast cancer survivors following axillary lymph node dissection (ALND). Compression of the affected limb is a mainstay of therapy. Surgical management of BCRL involves excision of excess fibroadipose tissue and physiologic procedures to improve fluid retention in the limb. Once lymphedema is established, the inflammatory cascade and fibrosis render the disease hard to reverse. The purpose of this review is to elucidate existing management strategies for prevention of breast cancer-related lymphedema. A literature search was conducted using PubMed, Ovid, Embase, and Scopus. Articles that included management strategies for prevention of BCRL were selected for review. Immediate lymphatic reconstruction (ILR) is a microsurgical technique that connects disrupted axillary lymphatic vessels to nearby veins by lymphovenous anastomoses at the time of ALND and has been shown to reduce rates of lymphedema from 30% to 4–12%. BCRL remains incurable. Immediate lymphatic reconstruction has emerged as a preventative strategy to reduce rates of lymphedema in breast cancer patients.

## 1. Introduction

Lymphedema is caused by lymphatic dysfunction and manifests as chronic limb swelling. Skin thickening, retention of protein-rich interstitial fluid, and deposition of subcutaneous fibroadipose tissue can result in progressive limb enlargement [1]. Lymphedema affects up to 250 million people worldwide, and it is associated with a significant impact on quality of life with a high burden of healthcare cost [2–5]. Morbidity from lymphedema includes recurrent cellulitis, pain, and impaired limb function [1,6,7]. Primary lymphedema constitutes 1% of cases and occurs from in-born errors in embryologic lymphatic system development [8,9]. Secondary lymphedema is the most common and is a consequence from injury to developed lymphatic channels. There is no cure for this life-long debilitating disease [1,6,10]. The objective of this scoping review is to synthetize existing literature on management strategies for prevention of breast cancer-related lymphedema.

Lymphedema affects 5 to 10 million people in the United States with 20,000 people diagnosed each year [2,11]. It is estimated that 20 to 40% of patients who undergo therapy for solid malignancies, such as breast cancer, melanoma, gynecological tumors, or sarcoma develop lymphedema [7,12]. One-third of breast cancer survivors who undergo axillary lymph node dissection (ALND) during surgical treatment diagnosis and/or treatment of the axilla acquire lymphedema [10,11,13,14]. ALND is performed for locally advanced breast cancer or biopsy-proven axillary lymph node metastases [15]. A randomized clinical trial has shown that ALND is recommended in patients undergoing breast conserving therapy who are found to have three or more positive lymph nodes for breast cancer from intraoperative sentinel lymph node biopsy [16,17]. Other indications for ALND include regional disease recurrence, residual lymph node disease burden after completion of neoadjuvant chemotherapy, and in patients who have a diagnosis of inflammatory breast cancer [17–21].

Physical characteristics of BCRL are based on the timeline of its course. In the initial stages of BCRL, progressive fluid accumulation is characterized by pitting edema of the affected extremity [22]. As the disease progresses into later stages, adipose deposition and fibrosis from chronic inflammation ensues leading to non-pitting edema [22]. The International Society of Lymphology utilizes a classification system to characterize lymphedema progression into four stages [23,24]. Stage 0 (subclinical) patients have a normal physical exam with abnormal lymphatic function on imaging [23,24]. Stage 1 patients present with limb edema that is improved with elevation [23,24]. Stage 2 patients develop pitting edema without improvement following limb elevation [23,24]. Stage 3 (non-pitting edema) occurs from chronic inflammation and fibroadipose disposition [25].

Diagnosis of lymphedema is based on clinical examination and imaging [24,26]. A Stemmer sign is a clinical exam that is positive when there is an inability to pinch the skin on the dorsum of the hand [27]. A positive Stemmer sign has a sensitivity of 97% and specificity of 95% [27]. Obesity has been shown to be an independent risk factor for development of secondary lymphedema after ALND [28]. A comparative study performed by Greene and colleagues showed that there are increased rates of infection, hospitalization, and larger extremities in obese patients, as defined by body mass index (BMI) greater than 30 kg/m^2^ when compared to normal weight patients with lymphedema [29].

Lymphoscintigraphy is used to confirm a diagnosis of lymphedema with 96% sensitivity and 100% specificity [26]. In suspected cases of BCRL, breast cancer patients receive intradermal injections of Technetium 99 mm sulfur colloid proximal to the second and fourth metacarpophalangeal joints of the affected and contralateral upper limbs. A positive study is considered when there is delayed transit (greater than 45 min) or absence of radioactive tracer to regional lymph nodes [30–32].

## 2. Results

The search yielded a total of 1083 articles of which 5 studies with a median follow-up of 25 months consisting of 1002 total patients with breast cancer were included for analysis (Table 1) [33–37]. ALND-only was performed in 57% (576/1002) of patients while ILR with ALND was performed in 43% (426/1002) of patients. From this cohort 91% (389/426) underwent adjuvant radiation therapy, 71% (302/426) neoadjuvant chemotherapy, and 47% (202/426) adjuvant chemotherapy. At 25-month follow-up, studies with ALND-only control groups had 26% (148/576) of patients developing BCRL compared to 16% (69/426) in their ILR with ALND experimental groups (*p* = 0.0003).

## 3. Discussion

BCRL occurs in up to 30% of patients following ALND [13,38,39]. Adjuvant radiation to the breast and axilla for breast cancer treatment has also been shown to be an independent risk factor for BCRL [40,41]. Up to 75% of patients who undergo ALND develop lymphedema within three years from the time of lymphatic channel disruption [42]. Accumulation of stagnant immune-rich fluid and protein leads to chronic inflammation, progressive limb enlargement, recurrent cellulitis, and functional limb impairment [1,43].

### 3.1. Surgical Management of Lymphedema

Surgical management of BCRL is guided based on disease progression and is divided into excisional and physiological procedures [44–46]. Excisional procedures decrease limb volume by removal of excess fibroadipose tissue [44–46]. Physiological procedures aim to improve lymph clearance of the affected limb. Excisional procedures for BCRL include skin/subcutaneous excision and suction-assisted lipectomy [44–47]. Skin and subcutaneous excision reduces limb volume by removal of subcutaneous fibroadipose tissue followed primary closure. Suctional-assisted lipectomy using liposuction techniques removes abnormally deposited suprafascial adipose tissue [47–49].

Vascularized lymph node transfer (VLNT) and lymphovenous bypass (LVB) are physiologic procedures for those with established postsurgical lymphedema [50]. Donor sites for lymph node free flap transfer include supraclavicular, axillary, lateral thoracic, deep inferior epigastric, superficial circumflex iliac, right gastroepiploic, and jejunal lymph nodes [51–56]. Omentum is most widely used for lymph node transfer in the United States without risk of donor site lymphedema [57,58]. When an abdominally based free flap breast reconstruction is performed concurrently with a VLNT, such as deep inferior epigastric artery perforator (DIEP) free flap, superficial groin lymph nodes by the superficial inferior epigastric artery, superficial circumflex iliac artery, or lymph nodes adjacent to the deep inferior epigastric artery can be used for lymph node transfer [54,59]. Reverse lymphatic mapping is required to harvest superficial groin lymph nodes to ensure that lymph nodes that are essential for lower limb drainage are not compromised so that donor site lymphedema does not occur [60]. Lymph nodes procured that are associated with the deep inferior epigastric artery do not require reverse lymphatic mapping [54]. An average of 2.8 lymph nodes are identified by the pedicle to the DIEP free flap near the junction of the medial and lateral row branches [61]. The vascularized lymph node flap for deep inferior epigastric artery nodes or superficial groin lymph nodes can remain in continuity with the DIEP flap for breast reconstruction as a “conjoined” flap. A prospective study evaluated 2-year outcomes following VLNT for patients with postsurgical lymphedema and BMI of 30 or less and showed that compression was no longer required in 34% of patients 2 years post operatively, as well as a 20% limb volume reduction and 27.5% bioimpedance score improvement, which demonstrated the safety and efficacy of VLNT [62].

Lymphovenous bypass is performed by anastomosis of lymphatic vessels to nearby venules in the lymphedematous limb [63–65]. Indocyanine green laser lymphangiography is used to identify lymphatic channels [63–65]. Subdermal lymphatics are anastomosed to adjacent venules through multiple skin incisions in the affected extremity that are planned based on the intraoperative lymphangiography [63–65]. Lymphatic vessels selected for LVB are typically less than 0.8 mm and this technique is referred to as “supermicrosurgery” [65]. The LVB is used to redirect lymph fluid from the injured regional lymph node basin by using lymphatic vessels to shunt lymph into the venous systemic circulation [65]. A 30% limb reduction has been demonstrated after physiologic procedures for lymphedema [44]. A systematic review on patency of lymphovenous anastomosis found a 52% patency rate at 5 months performed in canines [66].

### 3.2. Prevention of Lymphedema

**Figure 1.**
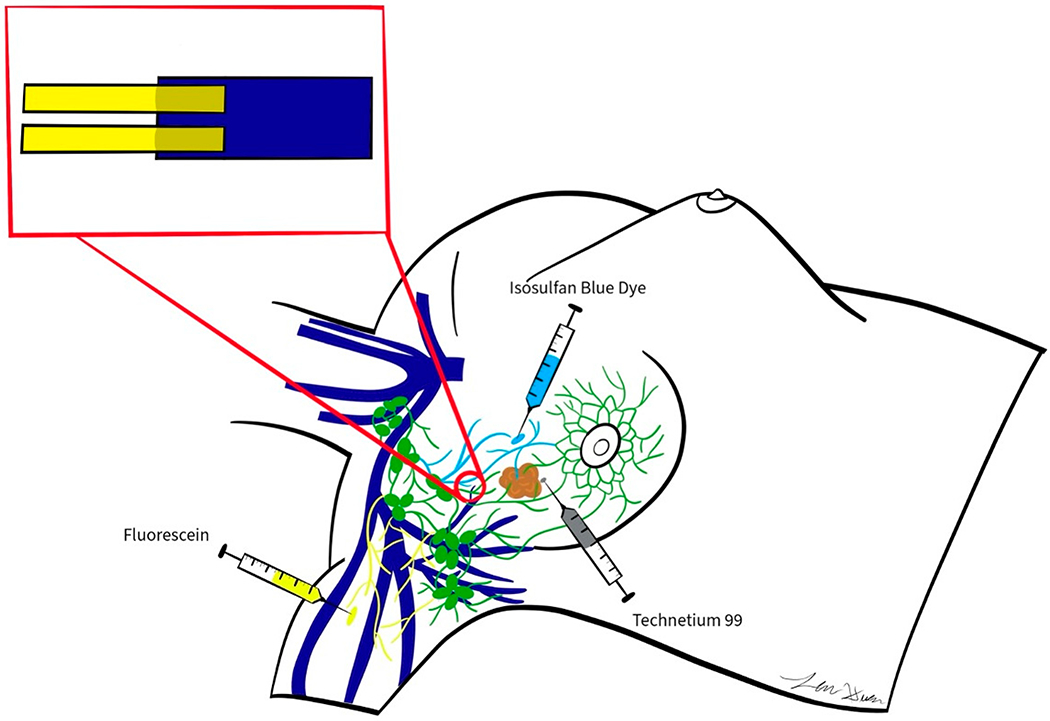
Illustration of immediate lymphatic reconstruction (ILR) following axillary lymph node dissection (ALND) for breast cancer. Disrupted afferent lymphatics that are critical for limb drainage are identified using isosulfan blue dye, indocyanine green dye, or fluorescent dye. When isosulfan blue dye is subcutaneously injected into the arm, transected afferent lymphatics in the axillary lymph node basin appear blue under an operating microscope. Transected afferent lymphatic channels can be anastomosed to nearby axillary or thoracodorsal vein branches in an end-to-end or end-to-side fashion. Meticulous dissection around the vein provides mobilization to achieve adequate length for a tension free lymphovenous anastomosis. Lymphatic channels are typically repaired using 11-0 nylon suture to a nearby vein branch. Injured lymphatic vessels may range between 0.3 and 0.8 mm and are significantly smaller than the vein branches. A sleeve technique can be performed in which smaller lymphatic channels are telescoped within the lumen of the vein.

Immediate lymphatic reconstruction (ILR), also known as Lymphatic Microsurgical Preventative Healing Approach (LYMPHA), is a preventative microsurgical technique to reduce the risk lymphedema [11,23,34,36,38,67–74] (Figure 1). Afferent axillary lymphatic vessels that are disrupted from lymph node dissection are anastomosed to nearby veins to restore lymph flow back into the systemic circulation. ILR was first described by Boccardo and colleagues, who reported a lymphedema rate reduction to 4% compared to a 30% rate in control patients who underwent ALND for breast cancer [38].

In this review, the rate of BCRL was 16% when ILR was performed following ALND compared to 26% in studies that performed ALND without ILR as a control group at 25-month follow-up [33–37]. BCRL rates have been reported by other groups that range from 0% to 12.5% after axillary lymph node dissection [10,38,67,69,74–79]. A systematic review demonstrated the incidence of lymphedema in patients who underwent ALND and received adjuvant radiation was 33.4% compared to a 10.3% incidence of lymphedema in patients when ILR was performed following ALND [10]. A previous systematic review of the literature demonstrated a 6.6% risk of BCRL in those undergoing ALND with ILR compared to a 30.5% lymphedema rate in patients who had ALND without ILR [70]. In a retrospective study of 148 breast cancer patients who underwent mastectomy, immediate breast reconstruction, and axillary node surgery, an intraoperative frozen section of sentinel/targeted nodes extended operative time by approximately 80 min in patients undergoing mastectomy with breast reconstruction and conversion of sentinel lymph node biopsy to ALND with ILR [80].

ILR is typically performed immediately following ALND [68–70,81,82]. Disrupted afferent lymphatics, which are critical for limb drainage, are visualized with an operating microscope using dye or fluorescent substances including isosulfan blue, indocynanine green (ICG), or fluorescein dye [69,73,83].

Axillary or thoracodorsal vein branches that are adjacent to the transected afferent lymphatic channel are selected for immediate lymphovenous anastomosis. Meticulous dissection around the vein provides mobilization to achieve adequate length for a tension free lymphovenous anastomosis [69,70]. Lymphatic channels are typically repaired using 11-0 nylon suture to a nearby vein branch [70]. An end-to-side or end-to-end anastomosis can be performed between an adequately sized matched lymphatic channel to a vein [70]. However, more commonly, injured lymphatic vessels range between 0.3 and 0.8 mm and are significantly smaller than the vein branches [84]. A sleeve technique can be performed in which smaller lymphatic channels are telescoped within the lumen of the vein [68–70]. Multiple lymphatic channels can be anastomosed using the sleeve technique [68–70]. In breast cancer patients who undergo ILR, an average of 3.5 (range 2 to 5) lymphovenous anastomoses are performed [71,84]. If lymphatic channels are not in close proximity for tension free anastomosis with the vein, further mobilization of the vein can be performed, or a vein graft can be used to link the vein to the lymphatics [72]. The vein graft can be procured from the axillary site if a suitable vein is found and expendable. Alternatively, vein graft can be harvested from the lower extremity [72]. A 9-0 to 11-0 nylon “U-Stich” suture is placed from the vein adventitia through the vein lumen and into the lymphatic lumen. Then the suture is driven back through the vein lumen and adventitia [71]. When the suture is tied, the disrupted lymphatic channels are secured into the vein lumen. Sutures can then be placed between the vein and the perilymphatic adipose tissue for further reinforcement of the anastomosis. The U-stich is then removed and lumen patency is confirmed with ICG or fluorescein lymphangiography [85]. Fibrin glue can be applied to further stabilize the anastomosis [86].

Axillary reverse mapping may be concomitantly performed if the breast surgical oncologist uses dye to identify axillary lymph nodes prior to ALND [87]. A 1% concentration of isosulfan blue containing 3 to 5 mL of dye may be injected into the brachial fascia and the arm is thoroughly massaged to promote dye uptake [87,88]. A surgical microscope is used to inspect the axillary lymph node basin after ALND [67,80,89]. In the case of using isosulfan blue, disrupted lymphatic vessels will appear blue under the surgical microscope. An alternative approach to identify disrupted lymphatics real-time is near-infrared laser lymphangiography using ICG or fluorescein with a 560 nm microscope filter [90–92].

**Figure 2.**
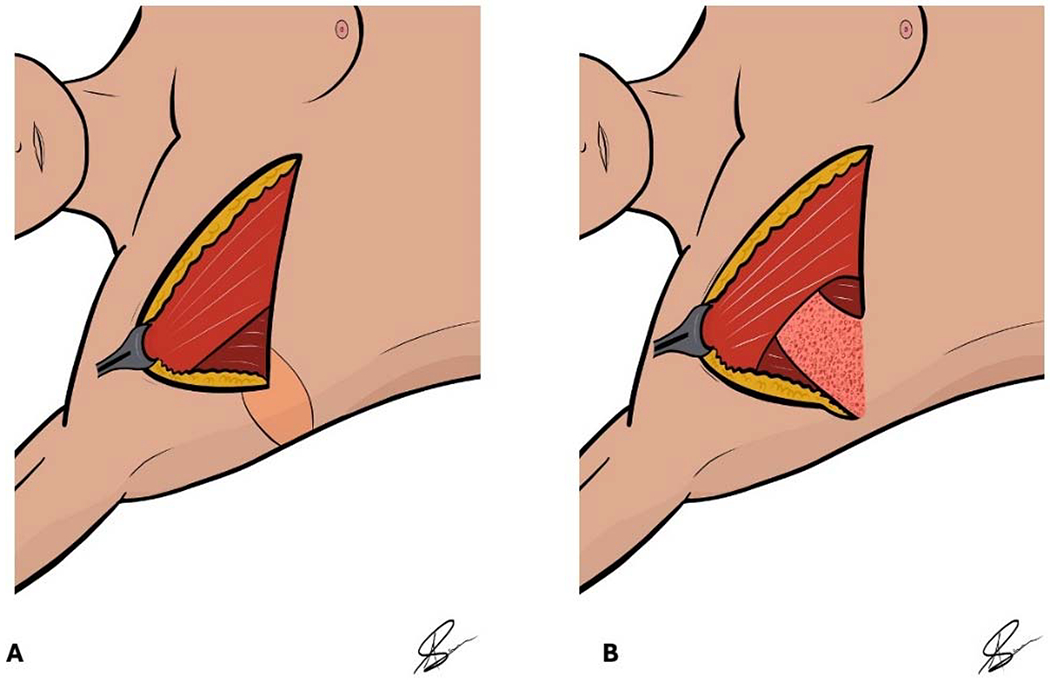
Prophylactic buried dermal flap illustration. (**A**) De-epithelization of the inferolateral mastectomy incision skin edge is performed. (**B**) A prophylactic buried dermal flap is transposed into the axillary dead space and over the ILR postmastectomy.

Skin and subcutaneous tissue that is typically a standing cone deformity lateral to the ALND incision may be used as a de-epithelialized buried dermal flap transposed into the axillary space [93]. A buried dermal flap may be an accessory procedure to ILR during axillary reconstruction (Figure 2) [93]. The buried dermal flap obliterates the dead space after ALND without significantly increasing operative time [93]. Vascularized lymph node transplant (VLNT) or pedicled axial flap like thoracodorsal artery perforator flap is an alternative approach to deliver soft tissue to the axilla after ALND [62,94]. However, an additional surgical site is required to harvest lymph nodes during VLNT, which may contribute to donor site morbidity [94]. A study of 13 patients who underwent ILR with vascularized omental lymph node transplant reported 37.5% (3/8) of patients had reduced shoulder abduction [94]. Greater complexity and operative time may hinder the wide adaptation as a routine prophylactic procedure used after axillary dissection.

Post-operative seroma formation is common after ALND [95–97]. Preventative measures including the use of quilting sutures and synthetic glue or fibrin sealants to close axillary dead space may reduce post-operative seroma rates and transient limb swelling [95–97]. A study of 100 patients who underwent closure of axillary dead space with quilting sutures termed the Chippendale Technique found a 50% reduction in seroma rates compared to the control group [96].

### 3.3. Experimental Therapies for Lymphedema

Evidence on experimental therapies have been assessed in preclinical models using mice. The murine tail is the most commonly used model to study secondary lymphedema [98–102]. Tacrolimus administered topically using the mouse tail model has shown improved lymphatic function with increased lymphatic collecting vessel contraction frequency [101]. Leukotriene B_4_ antagonism in the mouse tail lymphedema model has shown restoration of lymphatic architecture and improved lymphatic function, suggesting potential as drug target pathway for secondary lymphedema [102]. Focal delivery of *Prox1* (a master regulator of lymphangiogensis) using tissue nanotransfection technology (TNT) in the murine tail model has been studied to experimentally prevent lymphedema [100]. TNT uses a direct, transcutaneous nonviral vector gene delivery by way of a chip with nanochannel poration stimulated by a rapid focused electric field [100]. Mice treated with *Prox1* had less tail swelling and greater lymphatic clearance on lymphangiography compared to the control group [100]. The mouse hindlimb model is an alternative and potentially more clinically translatable method to study lymphedema [103]. However, inconsistencies including the use of radiation may contribute to the hindlimb model being less widely adapted [103]. Treatment using 9-cis retinoic acid intraperitoneally injected in the mouse hindlimb model has shown greater lymphatic clearance and less paw swelling compared to a non-operated, non-irradiated control limb [103]. A clinical trial of 15 BCRL patients used human recombinant vascular endothelial growth factor C (VEGF-C) with vascularized lymph node transfer followed by limb compression found a 46% reduction in limb circumference at 1-year follow-up [104]. A retrospective study of 17 BCRL patients who received oral doxycycline for 6 weeks found higher quality of life scores on a validated lymphedema patient-reported outcome instrument, although there was no difference in limb circumference between group at 17-week follow-up [105]. Larger studies with longterm follow-up typically of at least 2–3 years may be required to elucidate the impact of these clinical trials.

## 4. Methods

**Figure 3.**
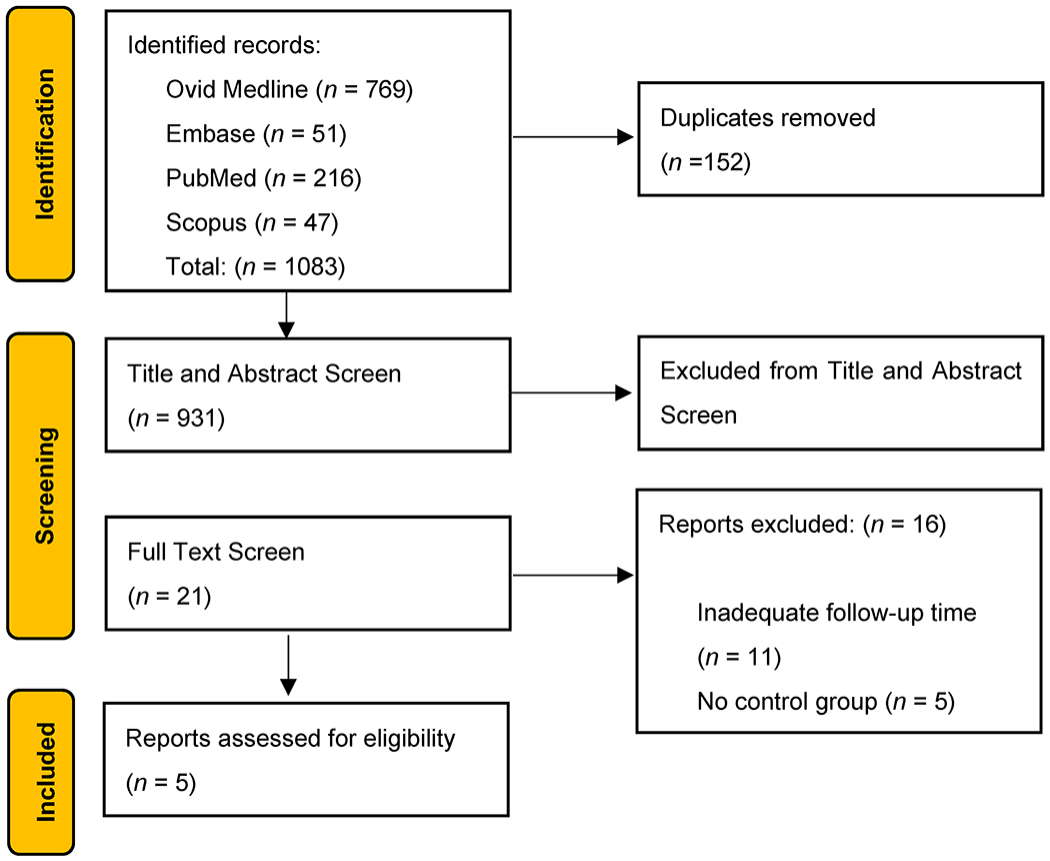
Preferred Reporting Items for Systematic reviews and Meta-Analyses extension for Scoping Reviews (PRISMA-ScR) Diagram Screening Process.

A systematic search was conducted using PubMed, Scopus, Ovid, and Embase databases. Original articles evaluating breast cancer patients who underwent ILR with ALND and ALND only control groups that had follow-up of at least 18 months were included. The mean onset of lymphedema typically occurs 9 months after axillary dissection [106]. Two independent authors screened titles and abstracts for relevance to select studies for review. Studies that were not written in the English language were excluded. Statistical analyses were performed using IBM SPSS Version 29 (IBM Corporation, Armonk, NY, USA). A *p*-value < 0.05 was considered statistically significant. The results of our search strategy, screening process, and full-text selection were reported through a Preferred Reporting Items for Systematic Review and Meta-Analyses (PRISMA) extension for a scoping review diagram (Figure 3) [107].

## 5. Limitations

There is a limited body of evidence on the clinical long-term outcomes of ILR [35,108]. A randomized prospective study of 144 patients with preliminary findings of ILR performed after ALND in breast cancer patients showed a significant reduction in BCRL to 9.5% compared to 32% in the control group 1 year post operatively [34]. A retrospective cohort study of 45 patients who received ILR showed that there was no significant difference in lymphedema incidence compared to a control group at the 4-year follow-up [108]. A prospective study of 230 patients randomized to ALND with ILR compared ALND without ILR found no difference in rates of BCRL through limb volume measurements in the two cohorts 3 years post operatively [35]. The discrepancy between studies may be related to how lymphedema is defined. Some patients may have earlier post-operative swelling that resolves. The need for studies on long-term outcomes following ILR are critical due to the delayed onset of lymphedema symptoms.

## 6. Conclusions

There is no cure for breast cancer-related lymphedema. Axillary lymph node dissection, delivery of adjuvant radiation, and obesity are independent risk factors for development of BCRL. ILR can be used prophylactically following ALND to reduce the long-term risk of BCRL incidence in patients, although further studies are required to further elucidate the effect of ILR on BCRL risk.

## Figures and Tables

**Table 1.** Included studies evaluating breast cancer patients who underwent immediate lymphatic reconstruction with axillary lymph node dissection (ALND) compared to a control group (ALND).

Ref.	Follow up Time (mo); (Mean/median)	Total Patients	ALND Only (*n* =)	ALND + ILR (*n* =)	Neoadjuvant Chemo (*n* =)	Adjuvant Chemo (*n* =)	Adjuvant Radiation (*n* =)	Control Cumulative BCRL (*n* =)	Experimental Cumulative BCRL (*n* =)	Control BCRL (%)	Experimental BCRL (%)
[33]	3 to 51; (23/_)	370	278	92	73	54	88	56	8	20.1	8.7
[34]	12 to 24; (_/18)	99	49	50	42	28	45	16	4	32.7	8
[35]	6 to 36; (_/29)	230	99	131	79	33	119	24	39	24.2	29.8
[36]	>24; (_/_)	171	94	77	72	29	70	36	8	38.3	10.4
[37]	3 to 60; (_/30)	132	56	76	36	58	67	16	10	28.6	13.2

## Data Availability

The data supporting the findings are available from the corresponding author upon reasonable request.
